# Surveillance of avian influenza viruses in South Korea between 2012 and 2014

**DOI:** 10.1186/s12985-017-0711-y

**Published:** 2017-03-14

**Authors:** Eun-Kyoung Lee, Hyun-Mi Kang, Byung-Min Song, Yu-NA Lee, Gyeong-Beum Heo, Hee-Soo Lee, Youn-Jeong Lee, Jae-Hong Kim

**Affiliations:** 1 Avian Disease Division, Animal and Plant Quarantine Agency, 177, Hyeoksin 8-ro, Gimcheon-si, Gyeongsangbuk-do 39660 Republic of Korea; 2 College of Veterinary Medicine, Seoul National University, 1, Gwanak-ro, Gwanak-gu, Seoul, 08826 Republic of Korea

**Keywords:** Avian influenza virus, Surveillance, Domestic poultry, LBM, Wild bird

## Abstract

**Background:**

National surveillance of avian influenza virus (AIV) in South Korea has been annually conducted for the early detection of AIV and responses to the introduction of highly pathogenic avian influenza (HPAI) virus. In this study, we report on a nationwide surveillance study of AIV in domestic poultry and wild birds in South Korea between 2012 and 2014.

**Methods:**

During the surveillance programs between 2012 and 2014, 141,560 samples were collected. Of these, 102,199 were from poultry farms, 8215 were from LBMs, and 31,146 were from wild bird habitats. The virus isolation was performed by inoculation of embryonated chicken eggs and AIV isolates were detected using hemagglutination assay. For subtying of AIV, the hemagglutinin and neuraminidase genes were confirmed by sequencing. Phylogenetic analysis of the H5 subtypes was performed using 28 H5 AIV isolates.

**Results:**

Between 2012 and 2014, a total of 819 AIV were isolated from 141,560 samples. Virus isolation rates for AIV were 0.6, 0.4, 0.1, and 2.7% in wild birds (*n* = 202), domestic ducks (*n* = 387), minor poultry (*n* = 11), and the live bird market (LBM) (*n* = 219), respectively. In wild birds, various subtypes were found including H1–H7 and H9–H13. The major subtypes were H5 (*n* = 48, 23.9%: N3 (*n* = 4) and N8 (*n* = 44)), H4 (*n* = 39, 19.4%), and H1 (*n* = 29, 14.4%). In domestic poultry, mainly ducks, the H5N8 (*n* = 275, 59.3%), H3 (*n* = 30, 17.2%), and H6 (*n* = 53, 11.4%) subtypes were predominantly found. The most frequently detected subtypes in LBM, primarily Korean native chicken, were H9 (*n* = 169, 77.2%). H3 (*n* = 10, 4%) and H6 (*n* = 30, 13.7%) were also isolated in LBM. Overall, the prevalence of AIV was found to be higher between winter and spring and in western parts of South Korea. The unusual high prevalence of the H5 subtype of AIV was due to the large scale outbreak of H5N8 HPAI in wild birds and domestic poultry in 2014.

**Conclusions:**

Enhanced surveillance and application of effective control measures in wild birds and domestic poultry, including LBM, should be implemented to control AI and eradicate HPAI.

**Electronic supplementary material:**

The online version of this article (doi:10.1186/s12985-017-0711-y) contains supplementary material, which is available to authorized users.

## Background

Avian influenza (AI) is a highly contagious viral disease that affects various avian species. The causative agent is avian influenza virus (AIV), which can be divided into subtypes based on the serogrouping of 16 hemagglutinin and 9 neuraminidase genes [1]. AIVs have a worldwide distribution in wild and domestic poultry and are broadly classified as low pathogenic avian influenza (LPAI) and highly pathogenic avian influenza (HPAI) virus. Highly pathogenic strains can cause high rates of and progressive mortality in commercial poultry flocks. Furthermore, they can lead to large outbreaks with severe economic consequences to the poultry industry of affected countries [2].

HPAI H5N1 virus was first isolated from sick geese in the Guangdong province in Hong Kong in 1996 and caused lethal outbreaks in poultry and humans in Hong Kong in 1997 [3]. Since re-emergence of the H5N1 virus was reported in 2003, AIVs have spread to over 70 countries across Asia, the Middle East, Europe, and Africa [4] and to numerous mammalian species including humans, resulting in 850 cases of infection and 449 deaths as of May 2016 [5]. With a threat to public health, H5N1 has caused major losses in poultry flocks in affected countries through either mortality or preventive culling.

In South Korea, there have been four outbreaks of H5N1 HPAI between 2003 and 2011 [6–9]. The fifth HPAI outbreak was reported in 2014 and was the first involving the H5N8 strain [10]. The H5N8 virus rapidly spread among western provinces where densities of overwintering waterfowl and domestic ducks are higher [11]. In late 2014, H5N8 outbreaks were reported in Europe and East Asia. Concurrently, this virus lineage was detected in North America [12, 13]. Three genetically distinct subgroups emerged and subsequently spread along different flyways during fall 2014 into Europe, North America, and East Asia [14]. In South Korea, H5N8 may have been reintroduced via bird migration in the winter season of 2014/2015 [11].

AIV surveillance in domestic poultry and wild birds is critical to our understanding of the persistence, transmission, and evolution of AI viruses. To monitor the incidence of AIV in domestic poultry and inform control programs, the Korean government launched nationwide surveillance programs in 2008. The surveillance strategy was designed to focus on early detection and responses to the introduction of HPAI

In the present study, we report on a nationwide surveillance study of AIV in domestic poultry and wild birds in South Korea between 2012 and 2014. The subtype and geographical distribution of AIV were determined, and their genetic relationship with neighboring countries was analyzed.

## Methods

### Surveillance

The national surveillance programs were initiated in 2008 in collaboration with the Ministry of Agriculture, Food and Rural Affairs (MAFRA), Animal and Plant Quarantine Agency (QIA), and Regional Animal Health offices to detect AIVs, particularly the H5 and H7 subtypes. The annual plans were nationally coordinated by MAFRA and QIA. Briefly, national surveillance targets divided into 6 categories such as wild bird (feces, carcass, captured), live bird markets (LBMs), domestic duck, domestic chicken, minor poultry, and imported raw materials of feed. All samples from each target were collected by regular intervals (Additional file 1: Table S1). Especially, we focused on wild bird, domestic duck farms and LBMs based on the accumulated data, which in mainly AIV were isolated, in previous studies [15–18].

Bird samples were collected by the Livestock Health Control Association (LHCA) or regional veterinary offices. The numbers of sample were dependent on surveillance target; for example, wild bird samples were collected 20–50 feces in one site of wild bird habitat. In the poultry farm, it was collected 20-birds orophryngeal swabs and 20 fecal samples in each house of farm. The samples were transported to regional veterinary laboratories in each province for AIV screening.

Positive samples for the H5 and H7 subtypes were detected using PCR and were shipped to the QIA promptly for presumptive findings, virus isolation, and pathogenicity. HA positive samples, which were negative for H5 and H7, were also analyzed for AIV subtyping and virus isolation by the QIA.

### Sample collection

During the surveillance programs between 2012 and 2014, 141,560 samples were collected. Of these, 102,199 were from poultry farms, 8215 were from LBMs, and 31,146 were from wild bird habitats. Sample collection was performed by the regional veterinary office or LHCA. Sampling site selection was based on the abundance of migratory birds, poultry population density and risks for transmission to farms. Oropharyngeal/cloacal swabs or fecal samples were collected from poultry farms, LBM, and wild bird habitats. Nationwide surveillance of AIV from the feces of wild birds in major migratory habitats was also conducted. Approximately 1000–2000 healthy migratory birds were captured per year by cannon netting, and oropharyngeal/cloacal swabs were collected from these animals. In LBMs, samples were collected from various poultry species such as ducks and chickens.

### Virus isolation

Oropharyngeal and cloacal swabs, fecal samples, and tissues from dead birds were suspended in antibiotic-treated phosphate buffered saline (PBS; p*H* 7.2) and inoculated into 9–11-day-old specific-pathogen-free (SPF) embryonated chicken eggs (ECEs; from SPAFAS, USA). Eggs were incubated at 37 °C for 4–5 days. The hemagglutination (HA) assay with chicken erythrocytes was used to detect AIV in allantoic fluid [19]. For subtyping of AIV, HA and neuraminidase (NA) genes of the AIV isolates were amplifiedwith gene-specific primers [20] using the One-Step RT-PCR kit (Qiagen, USA) and were confirmed by sequencing, which were identified by BLAST searches in the National Center for Biotechnology Information (NCBI, https://www.ncbi.nlm.nih.gov/nuccore) database. A barcoding system utilizing mitochondrial DNA of bird feces was employed to determine host species in AIV isolated from wild birds, as previously described [15].

### Phylogenetic analysis

Phylogenetic analysis of the H5 subtypes was performed using 28 H5 AIVs, which were selected among 329 isolates between 2012 and 2014 (Table 1). In this study, 10 H5 viruses, including the newly H5N8 HPAIVs isolated in LBM and H5 LPAIVs isolated from wild bird, were sequenced and analyzed together with 18 H5N8 representative isolates from previous published sequences [10, 11, 21]. Sequence information for the H5 AIV gene was obtained from NCBI and used to examine genetic relationships among AIVs isolated from South Korea and other countries (Additional file 2: Table S2). Sequences were aligned in MEGA using the MUSCLE algorithm. Maximum likelihood phylogenetic trees were constructed using the general time reversible (GTR) model with five gamma distributed heterogeneous substitution rates in MEGA 6.0 [22]. Nodal supports were assessed with 1000 bootstrap replicates.

**Table 1:** H5 Avian influenza viruses isolates in South Korea between 2012 and 2014

Virus isolate	Year	Surveillance targets	Species	Date	Subtype	Pathotype	Cleavage site	Reference	Accession no.
A/Baikal teal/Korea/Donglim3/2014	2014	Wild bird	Baikal teal	2014-01-17	H5N8	HPAI	PLRERRRKE/GLF	[10]	KJ413834
A/Baikal teal/Korea/H52/2014	2014	Wild bird	Baikal teal	2014-01-20	H5N8	HPAI	PLREKRRKE/GLF	[21]	KJ508961
A/Bean goose/Korea/H328/2014	2014	Wild bird	Bean goose	2014-02-01	H5N8	HPAI	PLRERRRKE/GLF	[21]	KJ509140
A/common teal/Korea/H455-30/2014	2014	Wild bird	common teal	2014-02-08	H5N8	HPAI	PLRERRRKE/GLF	[21]	KJ509156
A/gadwall/Korea/H1351/2014	2014	Wild bird	gadwall	2014-05-08	H5N8	HPAI	PLRERRRKE/GLF	[11]	EPI573208
A/spot-billed duck/Korea/H1981/2014	2014	Wild bird	spot-billed duck	2014-12-16	H5N8	HPAI	PLRERRRKE/GLF	[11]	EPI573236
A/mallard/Korea/H1991/2014	2014	Wild bird	mallard	2014-12-18	H5N8	HPAI	PLRERRRKE/GLF	[11]	EPI573237
A/breeder duck/Korea/Gochang1/2014	2014	Farm	breeder duck	2014-01-16	H5N8	HPAI	PLREKRRKE/GLF	[10]	KJ413842
A/broiler duck/Korea/Buan2/2014	2014	Farm	broiler duck	2014-01-17	H5N8	HPAI	PLRERRRKE/GLF	[10]	KJ413850
A/broiler duck/Korea/H651/2014	2014	Farm	broiler duck	2014-02-20	H5N8	HPAI	PLRERRRKE/GLF	[11]	EPI573196
A/chicken/Korea/H881/2014	2014	Farm	chicken	2014-03-06	H5N8	HPAI	PLRERRRKE/GLF	[11]	EPI573197
A/goose/Korea/H1296/2014	2014	Farm	domestic goose	2014-04-21	H5N8	HPAI	PLRERRRKE/GLF	[11]	EPI573204
A/broiler duck/Korea/H1582/2014	2014	Farm	broiler duck	2014-06-25	H5N8	HPAI	PLRERRRKE/GLF	[11]	EPI573214
A/broiler duck/Korea/H1685/2014	2014	Farm	broiler duck	2014-07-28	H5N8	HPAI	PLRERRRKE/GLF	[11]	EPI573217
A/broiler duck/Korea/H1731/2014	2014	Farm	broiler duck	2014-09-24	H5N8	HPAI	PLRERRRKE/GLF	[11]	EPI573221
A/breeder duck/Korea/H1752/2014	2014	Farm	breeder duck	2014-10-02	H5N8	HPAI	PLRERRRKE/GLF	[11]	EPI573227
A/broiler duck/Korea/H1840/2014	2014	Farm	broiler duck	2014-11-07	H5N8	HPAI	PLRERRRKE/GLF	[11]	EPI573232
A/mallard/Korea/H1924-6/2014	2014	Farm	domestic duck	2014-12-01	H5N8	HPAI	PLRERRRKE/GLF	[11]	EPI573239
A/domestic mallard/Korea/LBM176/2014	2014	LBM	domestic mallard	2014-07-26	H5N8	HPAI	PLRERRRKE/GLF	Present study	EPI888264
A/broiler duck/Korea/LBM281/2014	2014	LBM	broiler duck	2014-07-26	H5N8	HPAI	PLRERRRKE/GLF	Present study	EPI888265
A/KNC^a^/Korea/LBM282/2014	2014	LBM	KNC	2014-09-26	H5N8	HPAI	PLRERRRKE/GLF	Present study	EPI888266
A/KNC/Korea/LBM287/2014	2014	LBM	KNC	2014-09-27	H5N8	HPAI	PLRERRRKE/GLF	Present study	EPI888267
A/KNC/Korea/LBM288/2014	2014	LBM	KNC	2014-09-27	H5N8	HPAI	PLRERRRKE/GLF	Present study	EPI888268
A/KNC/Korea/LBM367/2014	2014	LBM	KNC	2014-12-23	H5N8	HPAI	PLRERRRKE/GLF	Present study	EPI888269
A/common teal/Korea/A40-1/2012	2012	wild bird	common teal	2012-12-18	H5N3	LPAI	PQRETR/GLF	Present study	EPI888270
A/wild bird feces/Korea/H598/2014	2014	wild bird	wild bird	2014-02-18	H5N3	LPAI	PQRETR/GLF	Present study	EPI888271
A/spot-billed duck/WA696/2014	2014	wild bird	spot-billed duck	2014-11-14	H5N3	LPAI	PQRETR/GLF	Present study	EPI888272
A/wild bird feces/Korea/H2016/2014	2014	wild bird	wild bird	2014-12-24	H5N3	LPAI	PQRETR/GLF	Present study	EPI888273

^a^ *KNC* Korean native chicken

## Results

### Prevalence of influenza viruses

**Fig. 1:**
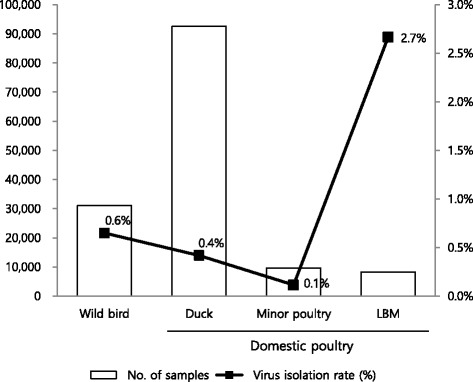
Prevalence of AIV in surveillance targets in South Korea between 2012 and 2014

From the 141,560 samples, 819 AIVs were isolated. The virus isolation rate was 0.6, 0.4, 0.1, and 2.7% in wild birds (*n* = 202), domestic duck (*n* = 387), minor poultry (*n* = 11), and LBMs (*n* = 219), respectively. AI surveillance of chicken farms was performed by monitoring clinical signs and serological analysis. HPAI outbreaks in chicken farms were not included in antigenic surveillance results. The highest virus isolation rate was observed in LBMs, which included samples from various species, primarily domestic ducks and Korean native chickens (Fig. 1).

**Fig. 2:**
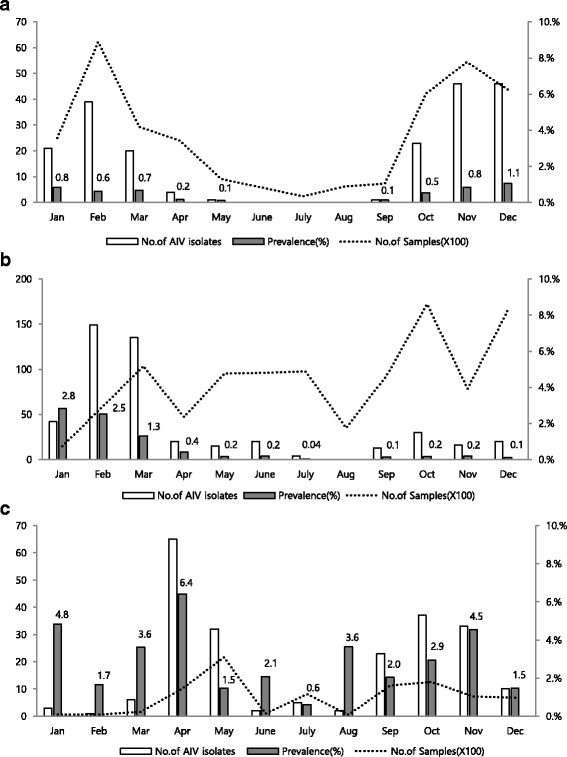
Monthly prevalence of AIV compared with sampling size between 2012 and 2014. The bars represent the number of AIV isolates during 2012–2014: **a** wild birds; **b** domestic poultry; and **c** live bird market. The lines represent the scaled number of samples. The left and right Y axes indicate the number of AIV isolates and samples, respectively. The X axis indicates months

In wild birds, the number of AIV isolates was higher in winter, which reflects an increasing wild bird population and sampling. The prevalence of AIV was lower in summer, reflecting the movement of migratory birds to their breeding grounds (Fig. 2a). AIV was isolated from domestic poultry throughout the year, with the exception of summer. The number of AIV isolates was increased in cumulative data during the study period (2012–2014) between February and March, which was caused by epidemics of the H5N8 and H3 subtypes (Fig. 2b and Additional file 3: Figure S1). In the LBMs, sampling was conducted on a yearly basis, and the prevalence of AIV was found to be most frequent in spring (April and May) and autumn (September and November), which indicated an increase in the poultry trade season (Fig. 2c).

### Frequency and geographical distribution of AIV subtypes

**Fig. 3:**
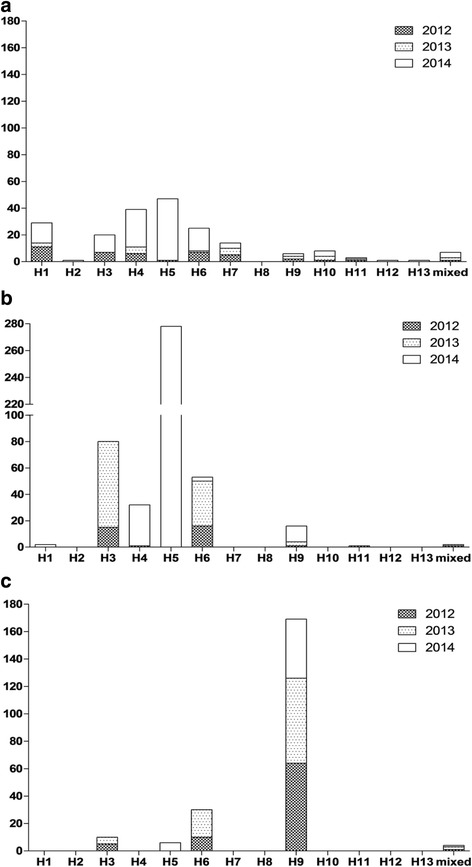
AIV HA subtypes in South Korea between 2012 and 2014. The bars represent the number of AIV isolates during 2012–2014: **a** wild bird; **b** domestic poultry; and **c** live bird markets

The AIV isolates from 2012 to 2014 were of various subtypes including H1–H7 and H9–H13 (Fig. 3). In wild birds, the major subtypes were H5 (*n* = 48, 23.9%), H4 (*n* = 39, 19.4%), and H1 (*n* = 29, 14.4%). H5 AIV was further classified into two NA subtypes: H5N3 (*n* = 4) and H5N8 (*n* = 44). In domestic poultry, H5 (*n* = 275, 59.3%), H3 (*n* = 80, 17.2%), and H6 (*n* = 53, 11.4%) were the most frequently detected subtypes. Since 2014, H5 subtypes in wild birds and domestic poultry have been predominant in accordance with an increase in HPAI H5N8 outbreaks. In LBMs, a total of 219 AIVs were isolated between 2012 and 2014. the H9 subtype (*n* = 169) was predominant and was mainly isolated from Korean native chickens, whereas, H3 (*n* = 10) and H6 (*n* = 30) subtypes were primarily isolated from duck species in LBMs. In 2014, the H5N8 subtype was isolated from Korean native chicken, broiler duck, and the domestic mandarin duck in LBMs (Table 1 and Fig. 3c).

**Fig. 4:**
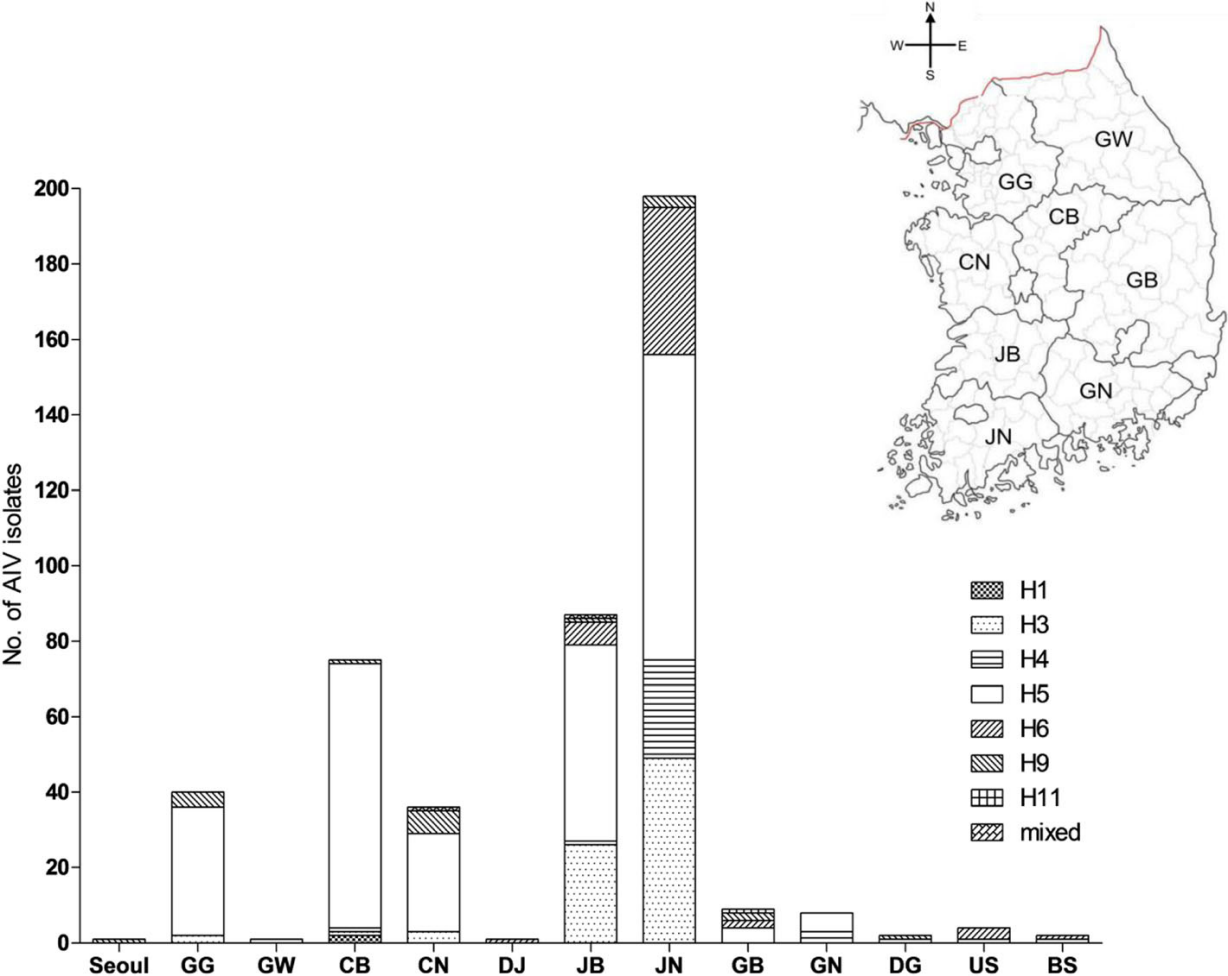
Geographical distribution of AIV HA subtypes in domestic poultry in South Korea between 2012 and 2014. Province abbreviations are as follows: GG, Gyeonggi; GW, Gangwon; CB, Chungbuk, CN, Chungnam; DJ, Daejeon; JB, Jeonbuk; JN, Jeonnam; GB, Gyeongbuk; GN, Gyeongnam; DG, Daegu; US, Ulsan; BS, Busan

AIV isolates from domestic poultry were largely found in western region of South Korea. The AIV distribution in domestic poultry was highest in Jeonnam (*n* = 198) followed by Jeonbuk (*n* = 87) (Fig. 4). In 2013, the H3 subtypes of AIV disseminated to broiler duck farms from the unique integrated breeder duck farms in Jeonnam and Jeonbuk (Fig. 4 and Additional file 3: Figure S1). The H5N8 subtype of AIV was also found to be predominant in western parts of South Korea (GG, CB, CN, GB, and GN), which has a high density of poultry farms (Fig. 4) [11].

### Phylogenetic analysis and molecular characterization

In this study, the HA of 10 H5 subtype isolates from wild birds (four) and LBMs (six) was newly sequenced, annotated, and confirmed. All of the H5N8 subtypes of AIV isolated from LBMs belonged to the H5 clade 2.3.4.4 and were confirmed as HPAI virus. These isolates have multiple basic amino acid sequences at the HA cleavage site, which is typical for HPAI viruses (Table 1). The four H5N3 subtypes originated from wild birds were LPAI viruses with a PQRETR amino acid motif at the HA cleavage site.

**Fig. 5:**
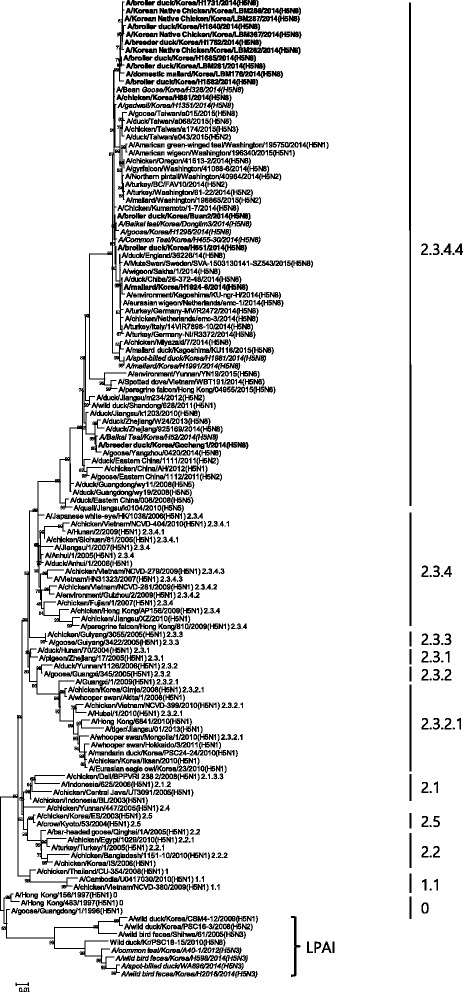
Phylogenetic tree for HA genes from H5 avian influenza viruses isolated in South Korea between 2012 and 2014. The H5 isolates from domestic poultry and wild birds are indicated in bold and italics, respectively. The vertical lines indicate HA gene clades

For phylogenetic analysis of the HA gene, a total of 28 representative H5 AIV isolates were selected and analyzed (Table 1). H5N8 HPAI viruses belonged to the H5 clade 2.3.4.4, and H5N3 LPAI viruses isolated from wild birds were clustered together into a group that was clearly distinct from H5 HPAI viruses. All of the HPAI viruses isolated from LBMs were closely related to H5 HPAI viruses that circulated in domestic duck farms in South Korea in 2014 (Fig. 5).

## Discussion

After the 2008 HPAI outbreak in South Korea, AIV surveillance systems were implemented to monitor the possible introduction of H5 or H7 HPAI. Nationwide AIV surveillance has resulted in the isolation of many subtypes of AIV in LBMs, duck farms, and wild birds. In the present study, we investigated AIV prevalence and subtypes in each surveillance target according to conduction of the national surveillance system.

The prevalence of AIV was 0.6% in the wild bird population and was consistent with the results of a previous surveillance study that demonstrated an overall prevalence of 0.8% in South Korea [15]. It seems that lower prevalence of AIV isolation was caused by using virus isolation method, not real-time RT-PCR. The number of AIV isolates was found to be higher between winter and spring. The AIV prevalence may have been affected by the overwintering season with a high population of migratory birds. It is hypothesized that the family *Anatidae*, which are considered to be the natural reservoir for AIV, migrate to Korea from September to March during wintering [11, 23].

AIV was isolated throughout the year in domestic poultry, with the exception of the month of August. Cumulative data demonstrated an increase in AIV isolates during February and March, which was caused by two factors. First, the unusual epidemics of H3 AIV subtypes occurred in duck farms related to specific integrated company in early 2013 (epidemiological investigation, Additional file 3: Figure S1). Second, H5N8 outbreaks affected the numbers of AIV infections during spring (Figs. 2 and 3). Between February and March 2014, AIV H5N8 (*n* = 157, 33.8%) was isolated from domestic poultry farms that primarily comprised ducks (*n* = 116). Ducks show few clinical signs and may serve as a potential carrier of AIV. Furthermore, small scale commercial duck farms belong to sector 3 production systems with low biosecurity and may play a key role in the distribution of AIV [24]. Intensifying biosecurity in duck farming is essential in controlling AI.

LBMs play an important role in mixing between various bird species and have been implicated in the spread of AIV [25]. In LBMs, the H9 subtype, mainly isolated from Korean native chickens, was predominant, whereas H3 and H6 subtypes were primarily isolated from duck species. In South Korea, the veterinary authority used stamping-out and compensation polices to eradicate the LPAI virus H9N2 subtype after the first outbreak in 1996; however, the H9N2 subtype has been endemic since 2000 [26]. In 2007, the veterinary authority permitted the use of the inactivated oil adjuvant H9N2 LPAI vaccine in commercial layers and broiler breeder chickens [27]. Since then, the prevalence of H9N2 virus has dramatically decreased in vaccinated chicken farms. However, these viruses have still been isolated primarily from Korean native chickens, which are reared in poorly managed farms and are primarily retailed via LBMs. Therefore, the H9 viruses were major subtypes in LBMs. In China, H5N1 virus and H7N9 virus, which cause human infection, are reassorted viruses with internal genes derived from H9N2 viruses circulating in domestic poultry, including LBM [28]. Furthermore, the novel H10N8 and H5N6 viruses in China emerged from LBMs, resulting in human infection [29, 30]. Although H9N2 isolates from South Korea were not found to be genetically related to these Chinese strains, continuous surveillance and tracing of the source of live birds in LBMs is essential to identify the emergence of novel AIVs and prevent potential risks to public health.

In 2014, H5N8 HPAIVs were detected in domestic poultry (*n* = 272) and wild birds (*n* = 44). With the exception of H5N8 subtypes, various subtypes were detected including H1, H3, H4, H6, and H7 in wild birds, whereas, H3, H4, and H6 subtypes were predominant in domestic poultry (mainly duck) (Fig. 3). These results are consistent with data from previous studies performed in South Korea, with the exception of the H5N8 subtype [15, 31, 32]. Enhanced active surveillance systems in wild birds and domestic poultry are essential to ensure early warning and reduce the circulation of AIVs in poultry population.

AIVs were mainly isolated from domestic poultry in western parts of South Korea. In 2014, the H5N8 outbreaks persisted in domestic duck farms in western provinces (Fig. 3). Geographically, the western and southern regions of South Korea were coincident with both HPAI outbreaks and densely populated poultry areas [11]. Furthermore, major reservoirs of migratory birds located in western parts of South Korea are resting and wintering sites for many migrating waterfowl from Northern Asia including Northern Russia, Mongolia, and Siberia. Enhanced surveillance of AIV should be performed in these risk areas for early detection and rapid application of control measures.

Phylogenetic analysis of H5 genes from 28 isolates between 2012 and 2014 revealed clustering of H5N8 HPAI viruses in clade 2.3.4.4. The HA gene of LPAI H5N3 viruses was found to originate from wild birds and could be clearly distinguished from the H5 HPAI viruses and clustered in the Eurasian lineages, which included previous Korean isolates (Fig. 5). H5 LPAI virus was only found in wild birds. AIV surveillance in wild birds is important for the application of early warning systems on the introduction of HPAI.

## Conclusion

For early detection of AIV and understanding potential risk factors in the spread of disease, AIV surveillance in South Korea is conducted on an annual basis. The present study demonstrated that most AIVs were isolated from wild birds, domestic ducks, and LBMs among 6 categories. Early warning systems in wild birds are very important for promoting prompt management actions to limit economic losses in poultry flock. Asymptomatic domestic ducks may serve as carriers of AIV. Therefore, enhanced surveillance by antigen detection is important in domestic duck farms. AIV prevalence of LBMs was higher than domestic poultry and wild bird. In addition, LBMs are the most important mixing point for various bird species and may be a major contributor in the spread of AIV and a source of human infections. Therefore, continued surveillance on wild birds, domestic poultry, and LBMs should be conducted and application of effective control measures which reduced circulation of AIV in poultry must be needed.

## Additional files

Additional file 1: Table S1. National active surveillace plan for avian influenza in 2014 in South Korea. (XLSX 14 kb)
Additional file 2: Table S2. HA sequence information of H5 viruses for phylogenetic analysis. (XLSX 18 kb)
Additional file 3: Figure S1. Monthly prevalence of domestic poultry about H5N8 subtype in 2014 and H3 subtype in 2013. (DOCX 16 kb)
