# Supplementary material for: Surveillance of avian influenza viruses in South Korea between 2012 and 2014
Source: Virol J. 2017 Mar 14;14:54. doi: 10.1186/s12985-017-0711-y (PMC5351195; doi:10.1186/s12985-017-0711-y)
Supplement: Additional file 3: Figure S1. — Monthly prevalence of domestic poultry about H5N8 subtype in 2014 and H3 subtype in 2013. (DOCX 16 kb) [file 12985_2017_711_MOESM3_ESM.docx]

**(A) H5N8 isolation in domestic poultry in 2014**

**(B) H3 isolation in domestic poultry in 2013**

**Supplementary figure. Monthly prevalence of domestic poultry about H5N8 subtype in 2014 and H3 subtype 2013.**
